# Reevaluation of Antioxidative Strategies for Birth Defect Prevention in Diabetic Pregnancies

**DOI:** 10.4172/2167-7956.1000145

**Published:** 2016-02-12

**Authors:** Zhiyong Zhao

**Affiliations:** Department of Obstetrics, Gynecology and Reproductive Sciences, University of Maryland School of Medicine, Baltimore, Maryland, USA

**Keywords:** Birth defects, Diabetic embryopathy, Oxidative stress, Antioxidant, Endoplasmic reticulum stress, Nitrosative stress, Intervention

## Abstract

Diabetes mellitus in early pregnancy is the most severe maternal disease that is counted for 10% of newborn infants with structural defects. With the rapid increases in the number of diabetic women in childbearing age, the birth defect rate is projected to elevate dramatically. Thus, prevention of embryonic malformations becomes an urgent task. Animal studies have revealed an involvement of oxidative stress in diabetic embryopathy and treatment with antioxidants can reduce embryonic abnormalities. However, the failure of clinical trials using free radical-scavenging antioxidants to alleviate oxidative stress-related diseases prompts researchers to reevaluate the strategy in birth defect prevention. Hyperglycemia also disturbs other intracellular homeostasis, generating aberrant conditions. Perturbed folding of newly synthesized proteins causes accumulation of unfolded and misfolded proteins in the lumen of the endoplasmic reticulum (ER). The ER under the stress activates signaling cascades, known as unfolded protein response, to suppress cell mitosis and/or trigger apoptosis. ER stress can be ameliorated by chemical chaperones, which promote protein folding. Hyperglycemia also stimulates the expression of nitric oxide (NO) synthase 2 (NOS2) to produce high levels of NO and reactive nitrogen species and augment protein nitrosylation and nitration, resulting in nitrosative stress. Inhibition of NOS2 using inhibitors has been demonstrated to reduce embryonic malformations in diabetic animals. Therefore, targeting ER and nitrosative stress conditions using specific agents to prevent birth defects in diabetic pregnancies warrant further investigations. Simultaneously targeting multiple stress conditions using combined agents is a potentially effective and feasible approach.

## Introduction

Congenital fetal anomalies are a major factor in perinatal mortality and infant disability. According to the World Health Organization, approximately two hundred seventy thousand newborn babies die each year from congenital abnormalities [1]. In the United States, where perinatal care is widely available, still nearly one hundred fifty babies are born yearly having at least one structural birth defect [2,3]. The cause of the developmental malformations is multifactorial. It includes genetic factors (e.g., chromosomal abnormalities, gene mutations), maternal diseases (e.g., infection, diabetes mellitus), medications (e.g., valproic acid, methotrexate), and environmental toxins (e.g., tobacco smoke, alcohol consumption, pollutants) [2,3].

Diabetes mellitus in early pregnancies is the most severe maternal disease to cause birth defects in newborn infants, in addition to other adverse pregnant outcomes including small for gestational age (growth restriction), large for gestational age (macrosomia), and perinatal demise (spontaneous abortion, stillbirth) [4–8]. Although aggressive glycemic control and perinatal care are available in the developed countries, the birth defect rates in diabetic pregnancies remain much higher than the background rate [5,6,9]. The diabetic complication in pregnancy, known as diabetic embryopathy, is becoming more serious because the number of women of childbearing age with diabetes has been rapidly increasing, along with the climbing numbers of diabetics in the population [10]. Thus, prevention of birth defects from diabetic pregnancies has become an urgent task for basic researchers and clinical care-takers.

## Oxidative Stress and Antioxidative Approaches for Intervention

**Figure 1:**
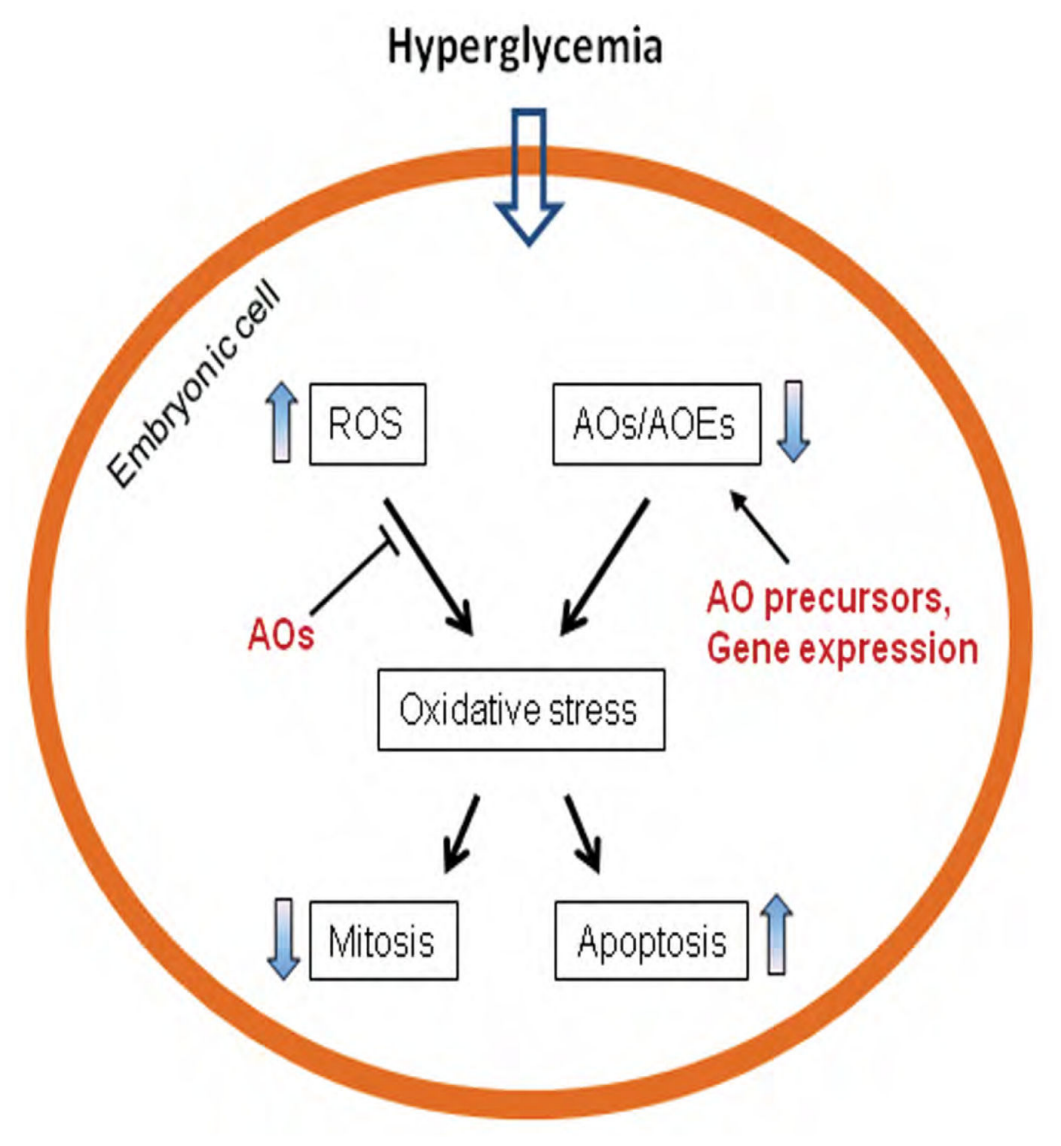
Oxidative stress in diabetic embryopathy. Hyperglycemia increases ROS (reactive oxygen species) and decreases AOs (antioxidants) and AOEs (antioxidative enzymes), leading to oxidative stress. To alleviate oxidative stress, AOs scavenge ROS, AO precursors increase the levels of endogenous AOs, and upregulation of AOE gene expression enhances antioxidative capacity.

It has been shown that maternal hyperglycemia alters mitochondrial morphology and function, leading to generate high levels of reactive oxygen species (ROS) [11,12]. Concurrently, hyperglycemia also reduces the levels of endogenous antioxidants, e.g., glutathione (GSH) and thioredoxin [13,14] and suppresses the expression and activity of antioxidative enzymes, e.g., superoxide dismutases (SODs), GSH peroxidases, and catalase [15–17]. This imbalance between levels of oxygen free radicals and antioxidative buffering, known as oxidative stress, augments oxidation of proteins, lipids, and DNA, leading to perturbation of intracellular signaling, organelle function, and gene regulation, and ultimately decreased cell proliferation and increased programmed cell death (Figure 1) [18,19].

The identification of oxidative stress in diabetic embryopathy led to development of strategies to reduce fetal abnormalities using antioxidants in animal models (Figure 1). Lipoic acid (LA or α-LA; an organosulfur compound) and vitamin C (VC; ascorbic acid) are hydrophilic free radical scavengers [20,21]. In diabetic pregnant animals, dietary supplementation of LA or VC has been shown to reduce embryonic malformation rate [22–25]. Vitamin E (VE; α-tocopherol) is a lipid-soluble antioxidant [26]. Treatment of diabetic pregnant animals with VE also lowers embryonic malformation rate [27–30]. N-acetylcysteine (NAC), a thiol-containing molecule, also can scavenge ROS [31]. In diabetic pregnant rats, treatment with NAC decreases neural tube and heart defects in the embryos [32,33]. Some naturally occurring phytochemicals possess antioxidant properties and have been tested to reduce embryonic defects in pregnant animals. For example, administration of resveratrol to diabetic pregnant rats reduces oxidative state and embryonic malformation rate [34,35].

Increasing the expression of endogenous antioxdative enzymes may enhance the intracellular defense against hyperglycemic insult. It has been shown that, in strains of rat that express higher levels of SOD and catalase, embryos of diabetic dams are resistant to hyperglycemic insult [36]. In diabetic transgenic mouse models, over expression of SOD1 in embryos reduces malformation rate [37]. Therefore, strategies to upregulate the expression of antioxidative enzymes to reduce embryonic abnormalities warrant development (Figure 1).

Although animal studies have shown that antioxidants can reduce developmental malformations in embryos, the enthusiasm about the application to humans has been dampened by the failure in multiple large scale trials using antioxidants (VC, VE) to treat similar diseases (preeclampsia, cardiovascular diseases) [38–41]. The reason(s) for the ineffectiveness of the antioxidants are not known. It is speculated that existence of other cellular stress conditions may be a factor [41].

## Targeting Endoplasmic Reticulum Stress

**Figure 2:**
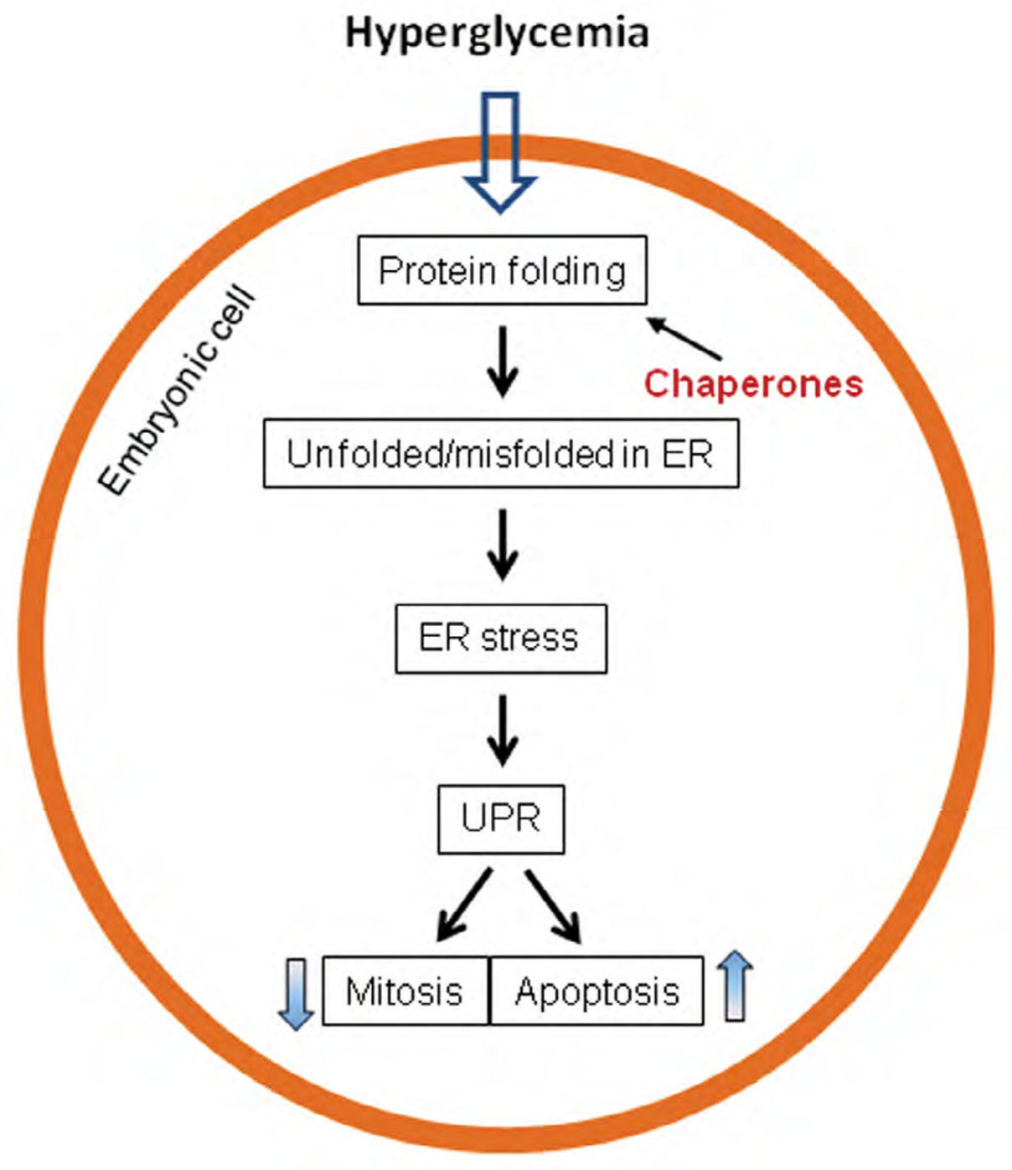
ER (endoplasmic reticulum) stress in diabetic embryopathy. Hyperglycemia disrupts protein folding and causes accumulation of unfolded or misfolded proteins in the lumen of ER. ER stress triggers UPR (unfolded protein response) signaling cascades to suppress cell mitosis and/or trigger apoptosis. ER stress can be ameliorated by chemical chaperones via promoting protein folding.

Indeed, stress in the endoplasmic reticulum (ER), exhibited by changes in specific factors (markers), in the embryos of diabetic animals has been observed [42–45]. Hyperglycemia disturbs the folding and processing of newly synthesized proteins. The unfolded and misfolded proteins are accumulated in the ER lumen, while the ER-associated protein degradation mechanism is impaired [46,47]. ER stress activates a number of molecular cascades, collectively known as the unfolded protein response (UPR), to upregulate chaperone proteins to resolve protein folding crisis, inhibit protein translation, suppress mitosis, and even trigger apoptosis (Figure 2) [48,49].

ER stress can be alleviated by enhancing protein folding, using chemical chaperones, including phenylbutyrate (PBA), ursodeoxycholic acid (UDCA), and taurine-conjugated derivative, tauroursodeoxycholic acid (TUDCA) (Figure 2). The chemical chaperones have been investigated in animal models and human diseases, such as cystic fibrosis and diabetes, and exhibited effectiveness in amelioration of the diseases [50–54]. PBA has been tested in mouse embryos cultured in high glucose and shown to reduce neural tube defects, making it a candidate for intervention in humans [45].

## Targeting Nitrosative Stress

**Figure 3:**
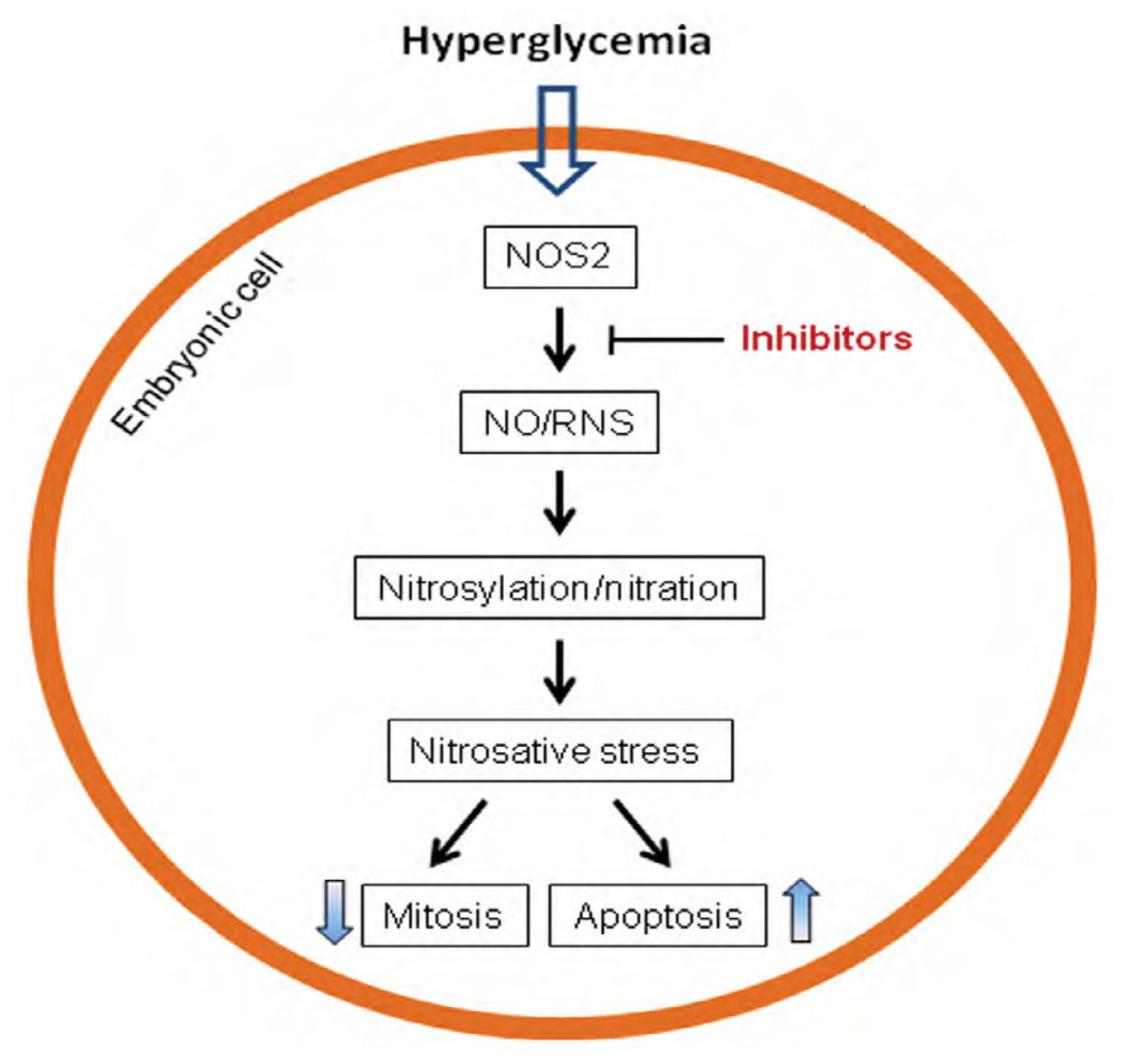
Nitrosative stress in diabetic embryopathy. Hyperglycemia stimulates the expression of NOS2 (nitric oxide synthase 2) to produce high levels of NO and RNS (reactive nitrogen species) and augment protein nitrosylation and nitration, resulting in nitrosative stress. Inhibition of NOS2 using inhibitors can ameliorate nitrosative stress.

In addition to dysfunction of the ER, maternal hyperglycemia also stimulates production of nitric oxide (NO) in the embryo, notably in the central nervous and cardiovascular systems [55,56]. NO is a reactive radical and also reacts with ROS to generate more cytotoxic derivatives, such as peroxynitrite, known as reactive nitrogen species (RNS) [57,58]. These free radicals augment protein S-nitrosylation and nitration, generating a condition known as nitrosative stress (Figure 3) [59,60]. Under such stress condition, protein activity is altered and organelle function is perturbed, leading decreased cell mitosis and increased apoptosis (Figure 3) [61–63].

NO is produced by members of the NO synthase (NOS) family, consisting of NOS1 (neuronal NOS or nNOS), NOS2 (inducible NOS or iNOS), and NOS3 (endothelial NOS or eNOS) [64–66]. nNOS and eNOS are constitutively expressed and do not vigorously respond to extracellular stimulation [67,68]. NOS2, on the other hand, actively responds to extracellular and intracellular stimulation with marked upregulation in expression and activity [69–71].

In the embryos of diabetic animals, NOS2 expression is increased, whereas the expression of NOS1 and NOS3 is decreased [72,73]. The significance of NOS2 in diabetic embryopathy has been demonstrated using a nos2 gene knockout animal model, showing significant decreases in malformation rates in the brain and heart in the embryos lacking the gene [56]. Efforts to target NOS2 to alleviate nitrosative stress have been made and shown promising results, which oral treatment of diabetic pregnant animals with NOS2 inhibitors reduces embryonic malformation rates (Figure 3) [43,56].

## New Strategies for Intervention in Diabetic Embryopathy

The failure of antioxidant trials apparently casts clouds over the effort to apply the similar approach to birth defect prevention; but it also opens opportunities for developing new strategies. Instead of scavenging free radicals, reinforcing the endogenous antioxidative capacity is an approach potentially to achieve effectiveness. This approach includes upregulation of antioxidative enzymes and replenishing of endogenous antioxidants.

NAC, a cysteine precursor, and folic acid (FA), a methionine precursor, can increase biosynthesis of GSH to enhance intracellular protection against ROS [74,75]. Treatment with FA has been shown to decrease abnormalities in the embryos of diabetic animals [76–78]. Retrospective clinical studies have shown the correlation of pre-conceptional FA intake with decreases in certain forms of fetal anomalies (neural tube defects) in diabetic pregnancies [79]. It is worth mentioning that the effect of FA on reduction of cardiac anomalies appears to be minimal [79,80]. The reason(s) for this are unknown, but it has been noticed that folate metabolism in diabetic pregnant women is not different from that in non-diabetic pregnant women [81].

In addition to the strategy of replenishing endogenous antioxidants, upregulation of antioxidative enzymes is also a powerful approach. This notion is supported by the experiments, in which overexpression of a superoxide dismutase in mouse embryos can decrease malformation rate in diabetic pregnancies [37,82]. Animal studies have shed lights onto these strategies. More work is needed to identify the effective and feasible agents to achieve the goals.

The embryos of diabetic pregnancies are under multiple cellular stress conditions (oxidative, ER, and nitrosative stresses). Each of the conditions can be intervened with pharmacological approaches *in vivo* (Figure 1). Targeting all the conditions simultaneously is potentially to be more effective to protect embryonic cells from maternal hyperglycemic insult. Treatments with cocktail of agents intervening different cellular signaling processes to reduce embryonic malformations in diabetic animals have shed lights on the feasibility of this approach [78,83–85]. With development of effective and safe agents for alleviating human intracellular stress conditions, prevention of birth defects in diabetic pregnancies will become reality using a combinational targeting approach.
